# DNA barcoding, species-specific PCR and real-time PCR techniques for the identification of six *Tribolium* pests of stored products

**DOI:** 10.1038/srep28494

**Published:** 2016-06-29

**Authors:** Tao Zhang, Yi-Jiao Wang, Wei Guo, Dan Luo, Yi Wu, Zuzana Kučerová, Václav Stejskal, George Opit, Yang Cao, Fu-Jun Li, Zhi-Hong Li

**Affiliations:** 1 College of Plant Protection, China Agricultural University, Beijing 100193, China; 2 Academy of State Administration of Grain, Beijing 100037, China; 3 Chinese Academy of Inspection and Quarantine, Beijing 100176, China; 4 Crop Research Institute, Drnovská 507, 161 06 Prague 6, Czech Republic; 5 Department of Entomology and Plant Pathology, 127 Noble Research Center, Oklahoma State University, Stillwater, OK 74078, USA

## Abstract

Flour beetles of the genus *Tribolium* Macleay (Coleoptera: Tenebrionidae) are important stored product pests in China and worldwide. They are often found or are intercepted in grain depots, flour mills, and entry-exit ports, etc. Traditionally, *Tribolium* species are identified according to the morphological characteristics of the adult. However, it is almost impossible to rapidly identify adult fragments and non-adult stages based on external morphological characteristics. Molecular techniques for the rapid and accurate identification of *Tribolium* species are required, particularly for pest monitoring and the quarantine of stored products pests. Here, we establish DNA barcoding, species-specific PCR, and real-time PCR techniques for the identification of six stored-product pest *Tribolium* species including *T. castaneum*, *T. confusum*, *T. destructor*, *T. madens*, *T. freemani* and *T. brevicornis*. We detected the mitochondrial DNA cytochrome oxidase subunit I (COI) barcodes for *Tribolium* from 18 geographic populations and 101 individuals, built a *Tribolium* DNA barcode library, and designed species-specific primers and TaqMan probes for the above six *Tribolium* species. The three techniques were applied to identify *Tribolium* collected from stored samples and samples captured from quarantine ports. The results demonstrated that three techniques were all able to identify the six species of *Tribolium* both rapidly and accurately.

Flour beetles of the genus *Tribolium* Macleay, 1825 (Coleoptera: Tenebrionidae) decrease the quality and quantity of stored products by introducing insect fragments and excrement^1^,^2^ as well as causing contamination with potential adverse effects to human health^3^. The genus *Tribolium* has 36 described species^4^, ten of which are stored products pests^5^. *T. castaneum* and *T. confusum* are major pests of processed grain and cereal products with cosmopolitan distribution, including P.R. China^6^, USA^7^ and Europe^8^, and five species of *Tribolium* (*T. destructor*, *T. audax* Halstead (1969), *T. madens*, *T. freemani*, and *T. anaphe* Hinton (1948)) are potentially serious pest species. The other *Tribolium* species are less harmful and include *T. brevicorne*, *T. parallelus* (Casey 1890), and *T. thusa* Hinton (1948).

Traditional methods for the identification of *Tribolium* species using the morphological characteristics of adults are common and widely used^9^. However, an individual *Tribolium* is small and many species are similar in appearance, particularly the non-adult stages including eggs, pupae and larvae. In routine stored-grain insect pest monitoring and port quarantine, it is difficult to identify species on the basis of external morphological characteristics alone. Recently, the rapid and accurate molecular identification of insect pests has become popular^10^,^11^,^12^. Therefore, the molecular identification of *Tribolium* species is an appropriate alternative to conventional taxonomy based on morphological characteristics^11^; moreover species-specific primers for *T. castaneum* and *T. confusum* have already been designed based on internal transcribed spacer (ITS) fragments encoded by rDNA and on mitochondrial cytochrome oxidase subunit I^1^. General PCR and PCR-RFLP analysis have been used to discriminate *T. destructor* from other species^13^. PCR-RFLP has been developed to distinguish *Tribolium* flour beetles based on a partial 28S rRNA gene sequence^14^. However, there are currently no correlative studies on the molecular identification of *Tribolium* species using DNA barcoding technology, species-specific PCR and real-time PCR.

This study focuses on six *Tribolium* species that are stored-product pests. The aim of the study was to establish molecular techniques to identify *Tribolium* species accurately, rapidly and practically using DNA barcoding, species-specific PCR, and real-time PCR.

## Materials and Methods

### Tribolium specimens

Cultures of six *Tribolium* species from 101 adult specimens including *T. castaneum*, *T. confusum*, *T. destructor*, *T. madens*, *T. freemani* and *T. brevicornis* were used in this study. Specimens were collected from the P. R. China (Henan, Guangxi, Guangdong, Xinjiang), the Czech Republic (Prague, Kyjov, Herink, Rakovník), France (Bordeaux), Croatia (Osijek), and the United States (Kansas). Original collection locations are listed in Table 1. These laboratory strains were reared in darkness at 27 °C and 75% relative humidity on a diet of powdered wheat germ. Voucher specimens, including adults, larvae and pupae, were kept in 100% ethanol and stored at −80 °C. Detailed specimen information is shown in Table 1.

### DNA extraction, PCR and COI sequencing

Total genomic DNA at least three *Tribolium* adults from different geographic population was extracted from the thorax of *Tribolium* adults using a commercial TIANamp Genomic DNA kit (TIANGEN, China) according to the manufacturer’s protocol. A pair of universal forward LCO1490 (5′-GGTCAACAAA TCATAAAGATATTGG-3′) and reverse HCO2198 (5′-TAAACTTCAGGGTGACCA AAAAATCA-3′) primers were used for COI amplification^15^. PCR was performed based on methods by Wang *et al*.^11^ and was modified for half volume reactions containing 12.5 μl MasterMix with loading dye, 10 μl sterilized distilled water, 1.5 μl extracted DNA (approximately 20 ng μL^−1^), and 0.5 μl forward and reverse primers (10 μM). The PCR protocol included an initial denaturing step at 94 °C for 3 min, followed by 35 cycles of 94 °C for 1 min, 52 °C for 1 min and 72 °C for 1 min with a final extension at 72 °C for 10 min. The reactions were performed on a Veriti TM 96-well Thermal Cycler (ABI, USA). The amplified DNA fragments were resolved on a 1.0% (w/v) agarose gel (1 × Tris Acetate-EDTA buffer), stained with ethidium bromide and visualized with a UV light (Gel Logic 212 PRO, Carestream Health, Inc.). DNA purification and bidirectional sequencing using the same amplification primers was commercially performed by Sangon Biotech (Shanghai) Co., Ltd. and Beijing Aoke Biotechnology Co., Ltd.

### Sequence assembly and analysis

Contig Express program was used to produce contigs from the forward and reverse reads of each COI amplicon and correct each read by looking at the chromatogram. Primer sequences from each contig were removed within the Contig Express program also. DNAMAN 7.0.2 software was used for DNA multiple sequences alignment. 110 amplicon sequences were aligned, among which 9 from GenBank and 101 from laboratory. Haplotypes were identified using the DnaSP v.5.1 software^16^. Pairwise genetic distances for COI genes were computed with the Kimura 2-parameter method (K2P). Neighbour-joining (NJ) phylogenetic trees were constructed in MEGA 6.0^17^, and distance histograms were generated with the online version of automatic barcode gap discovery (ABGD)^18^. All of the identified haplotypes were submitted to GenBank.

### Specific primer design, selection and sensitivity test

According to the variability of the partial COI gene sequences from six *Tribolium* species, suitable areas for designing species-specific primers were identified with Bioedit (version 7.2.0), and species-specific primers for *Tribolium* identification were designed with Primer Premier 5.0. Primer pairs were evaluated according to eight factors: (1) length between 18 bp to 30 bp; (2) absolute value of Delta G less than 9; (3) 3′-end contains one or more specific bases; (4) no distinct hairpin structure; (5) GC% from 30% to 70%; (6) primers for distinguishing different species; (7) false priming less than 100%; and (8) optimal annealing temperatures. All of the primers were synthesized by Sangon Biotech (Shanghai) Co., Ltd.

Specificity testing with each primer pair in the PCR assays was performed using 18 selected samples (Table 1). PCR amplification in a final reaction volume of 25 μl consisted of 12.5 μl MasterMix with dye, 10.5 μl ddH_2_O, 0.5 μl specific forward primer, 0.5 μl specific reverse primer and 1 μl template DNA. The PCR cycler conditions used were an initial denaturation at 94 °C for 3 min, followed by 35 cycles of 94 °C for 30 s, 54 °C for 30 s and 72 °C for 30 s with a final extension at 72 °C for 10 min. After separation by 1.5% agarose gel electrophoresis and staining in ethidium bromide, the products were confirmed under UV light (Gel Logic 212 PRO, Carestream Health, Inc.) and were sequenced in both directions by Beijing Aoke Biotechnology Co., Ltd.

Sensitivity testing with the selected six *Tribolium* species-specific primers was determined in PCR runs with a series of samples using decreasing DNA concentrations with the same primer concentration. The DNA concentrations used were 100, 10, 1, 0.1, 0.01 and 0.001 ng μl^−1^.

### TaqMan probe and real-time PCR primer design, selection and sensitivity test

According to the partial COI gene sequences of six *Tribolium* species, suitable areas for specific primers were identified by Bioedit (version 7.2.0). TaqMan probes and real-time PCR primers were designed with Beacon Designer 8.12. TaqMan probes were evaluated according to seven factors: (1) length between 18 bp and 30 bp; (2) C% more than G%, otherwise complementary use; (3) GC content between 30% and 80%; (4) no G bases at the 5′ end; (5) avoided repeats of the same type of bases, especially four G bases encoded together; (6) probe should be as close as possible to the primers; and (7) no complementary secondary structures or primers.

Real-time PCR primers were evaluated according to six factors: (1) the length of a pair of primers was no more than four bases, with single base primers between 18 bp and 30 bp; (2) GC content between 40% and 60%; (3) no A bases as the first base on the 3′ end; (4) avoided repeat of the same type of base, especially four G bases encoded together; (5) did not use three G or C bases in a row at the 3′ end; (6) no complimentary primers.

Real-time quantitative PCR reactions were processed in 96-well plates in the PCR amplifier (ABI7500) using commercial Premix Ex Taq (rr390A) according to the manufacturer’s protocol. PCR amplification in a final reaction volume of 20 μl contained 10 μl Premix Ex Taq (Probe qPCR, 2X), 0.4 μl specific forward primer, 0.4 μl specific reverse primer, 0.8 μl TaqMan probe, 7.4 μl ddH_2_O, and 1 μl template DNA. The PCR cycler conditions used were an initial denaturation at 95 °C for 30 s, followed by 35 cycles of 95 °C for 5 s, 60 °C for 34 s and with a final extension at 72 °C for 10 min.

Sensitivity testing of the selected TaqMan probes for the six *Tribolium* species was determined in PCR runs with a series of samples using decreasing DNA concentrations with the same primer concentration. The DNA concentration series was 100, 10, 1, 0.1, 0.01 and 0.001 ng μl^−1^. Three replicates of each treatment were tested.

## Results

DNA barcoding, species-specific PCR and real-time PCR accurately identified six stored-product pest *Tribolium* species including *T. castaneum*, *T. confusum*, *T. destructor*, *T. madens*, *T. freemani* and *T. brevicornis*.

### DNA barcoding, genetic divergence and phylogenetic analysis

A 658-bp long region of the mtDNA COI gene was amplified from 101 individual *Tribolium* beetles (Table 1) using a set of universal COI primers. Alignment of these sequences, and nine additional *Tribolium* sequences from GenBank (Table 2), revealed six haplotypes were identified for *T. castaneum*, one haplotype was observed for *T. confusum*, *T. destructor*, *T. madens*, *T. freemani* and *T. brevicornis*. The sequences of common haplotype from different geographic populations have been submitted to GenBank, obtain 24 accession number (Table 1). The DNA multiple sequence alignment using DNAMAN 7.0.2 software showed that four bases A, G, C, T average content of these sequences, 30.04%, 22.45%, 16.22%, 31.29% respectively, A + T content 61.33%. Base composition of *Tribolium* sequences in line with insect mitochondrial genes.

K2P model calculation results using MEGA 6.0 showed that intra- and inter-specific genetic distance exists obvious difference. The inter-specific K2P divergence of the six *Tribolium* species averaged 19.61%, ranging from 16.34% to 22.28%, such as genetic distance of *T. brevicornis* and *T. freemani* reached 22.28%, *T. castaneum* and *T. freemani* only 16.34%. The intra-specific K2P divergence ranged from 0.00% to 1.86%, with an average of 0.5% (Table 3). Specimens of *T. castaneum* were characterized by K2P divergence values up to 1.86%, but less than 2.0%. Intra-specific divergences of other *Tribolium* species were all less than 1.0%. All inter-specific divergence values were greater than intra-specific values, more than 39 times. A favourable DNA barcode should have a higher divergence among species than within species. In the case of COI, the suggested standard divergence threshold value is ten times (10×) the mean intra-specific variation^20^.

**Figure 1:**
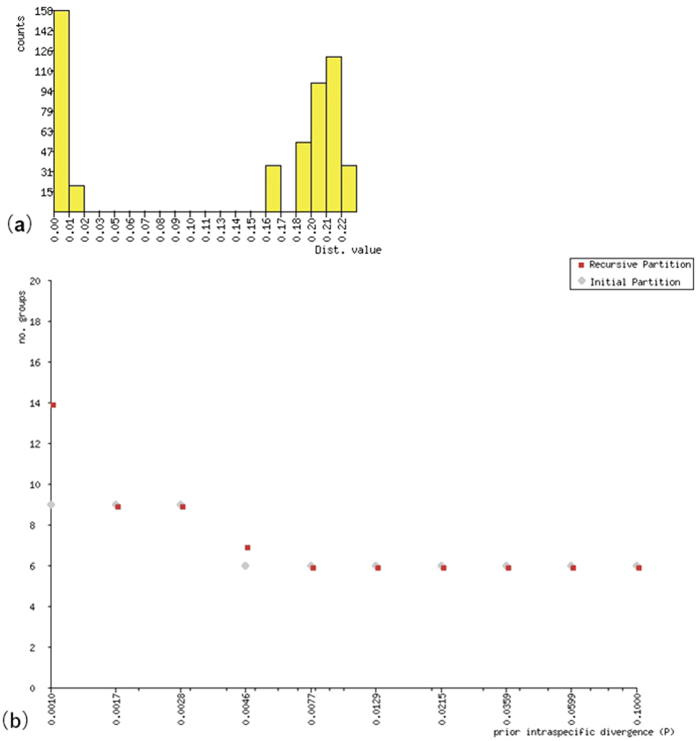
(**a,b**) The results of *Tribolium* sample analysis by ABGD. (**a**) Histogram of distances. (**b**) Automatic partition results for *Tribolium* taxa by ABGD.

The results of applying the ABGD algorithm to the COI data set are presented in Fig. 1. Distance values show a gap between the intra-specific and the inter-specific distances (Fig. 1a). The data set was partitioned into six groups when the prior assumption of maximum intra-specific divergence was set as high as 0.46% (Fig. 1b).

**Figure 2:**
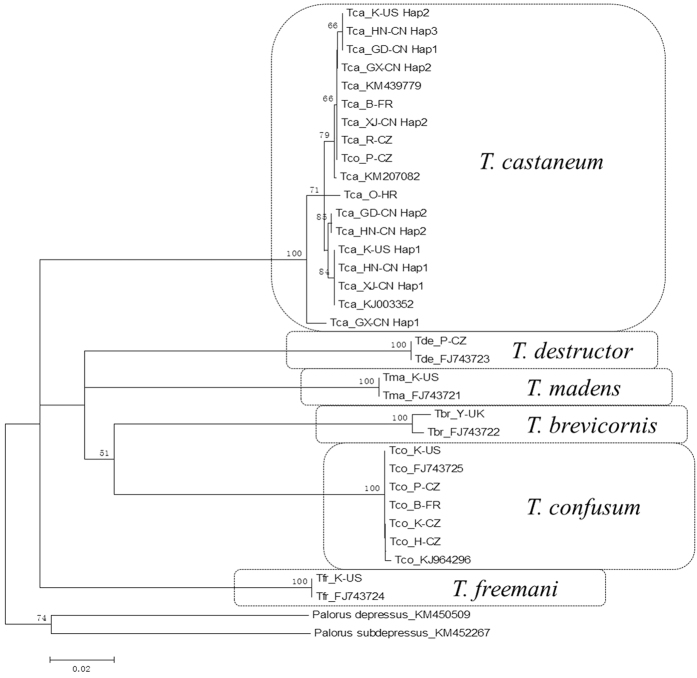
Neighbour-joining phylogenetic tree of *Tribolium* species based on the COI gene sequences.

The NJ tree grouped the six morphologically identified *Tribolium* species based on the COI gene sequences as well as the outgroup species *P. depressus* (GenBank submission number KM450509) and *P. subdepressus* (KM452267) (Fig. 2). The resulting trees showed a clear clade of six *Tribolium* species distinct from the outgroup clades. There was high bootstrap support (100%) for the terminal branches at the species level.

### Specific primer design, selection and sensitivity test

**Figure 3:**
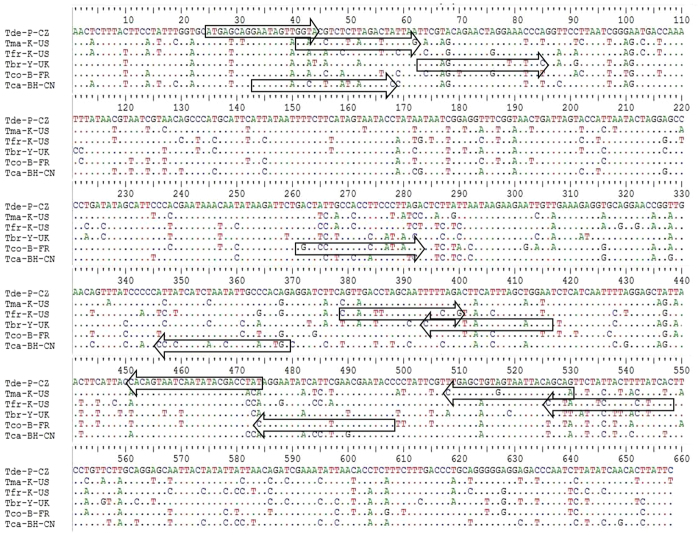
Aligned COI nucleotide sequences from six *Tribolium* species obtained in this study. Note: The sequences framed in the left arrow are the forward primers; the sequences framed in the right arrow are the reverse primers.

**Figure 4:**
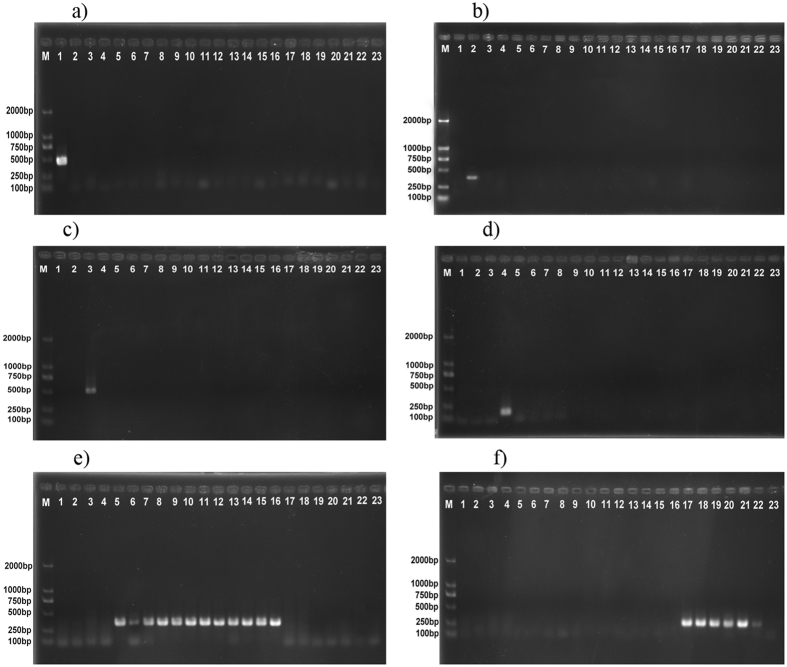
Gels from six PCR reactions validating the specificity of six *Tribolium* primer pairs. (**a**) Specific primers for *T. destructor*. (**b**) Specific primers for *T. brevicornis*. (**c**) Specific primers for *T. madens*. (**d**) Specific primers for *T. freeman*. (**e**) Specific primers for *T. castaneum*. (**f**) Specific primers for *T. confusum*. M: D2000 DNA Marker; 1: *T. destructor* (Prague); 2: *T. brevicornis* (York); 3: *T. madens* (Kansas); 4: *T. freemani* (Kansas); 5: *T. castaneum* (Prague); 6: *T. castaneum* (Rakovník); 7: *T. castaneum* (Osijek); 8: *T. castaneum* (Bordeaux); 9: *T. castaneum* (Kansas); 10,11: *T. castaneum* (guangxi); 12,13: *T. castaneum* (Xinjiang); 14: *T. castaneum* (Guangdong); 15: *T. castaneum* (Henan); 16: *T. confusum* (Prague); 17: *T. confusum* (Bordeaux); 18: *T. confusum* (Herink); 19: *T. confusum* (Kyjov); 20: *T. confusum* (Kansas); 21: Negative control (ddH2O).

One hundred and one partial COI gene sequences (658 bp) from six adult species in the genus *Tribolium* were used to develop an accurate and fast method for identifying these six *Tribolium* species (Fig. 3). The specificity tests of the designed primers were performed by uniplex PCR. Six primer pairs were selected for the reliable identification of six *Tribolium* species and are listed in Table 4. These primer pairs were designed to identify different *Tribolium* species regardless of life stage. The results clearly demonstrated that each primer pair produced a species-specific band without any nonspecific bands (Fig. 4).

**Figure 5:**
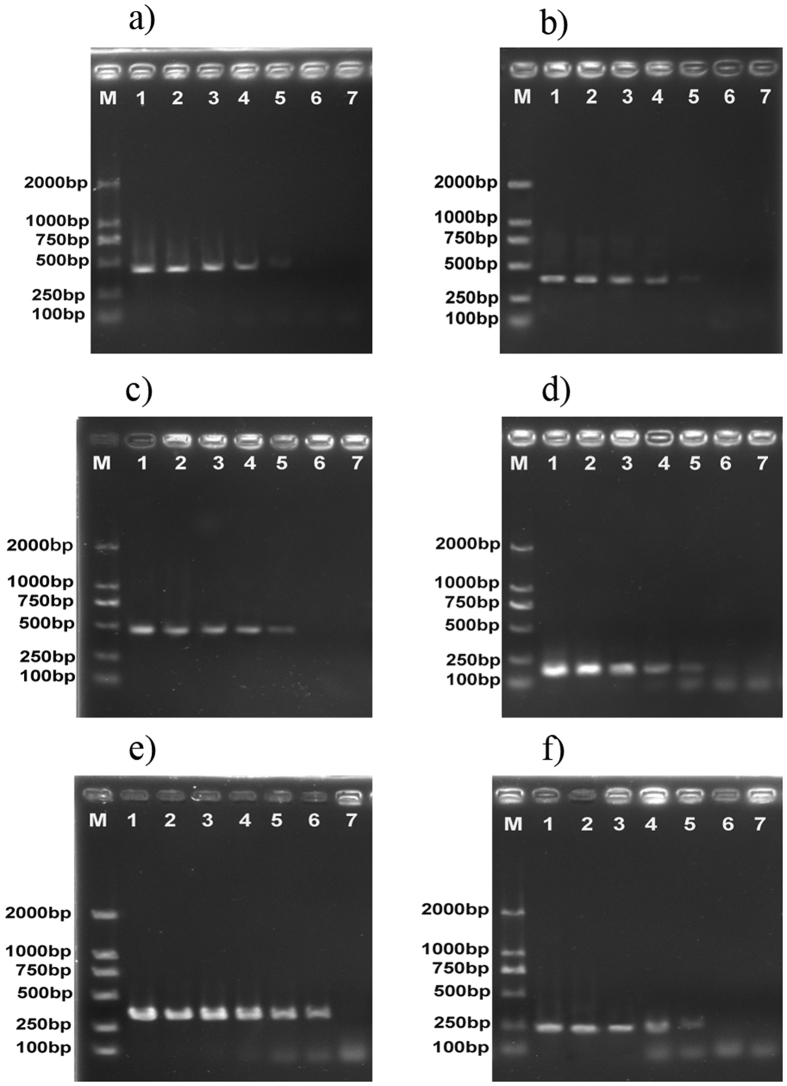
Sensitivity tests for six specific primer sets. (**a**) *T. destructor*. (**b**) T. brevicornis. (**c**) *T. madens*. (**d**) *T. freeman*. (**e**) *T. castaneum*. (**f**) *T. confusum*. The concentration of template DNA from lane 1 to lane 6 was 100, 10, 1, 0.1, 0.01, and 0.001 ng μl^−1^. Lane M: DNA Marker.

Sensitivity of a selected specific primer set for each of the six species was determined using one sample from each species. In all of the species, DNA concentrations of 100, 10, 1, and 0.1 ng μl^−1^ resulted in strong intensity bands (Fig. 5).

### TaqMan probe and real-time PCR primer design, selection and sensitivity test

**Figure 6:**
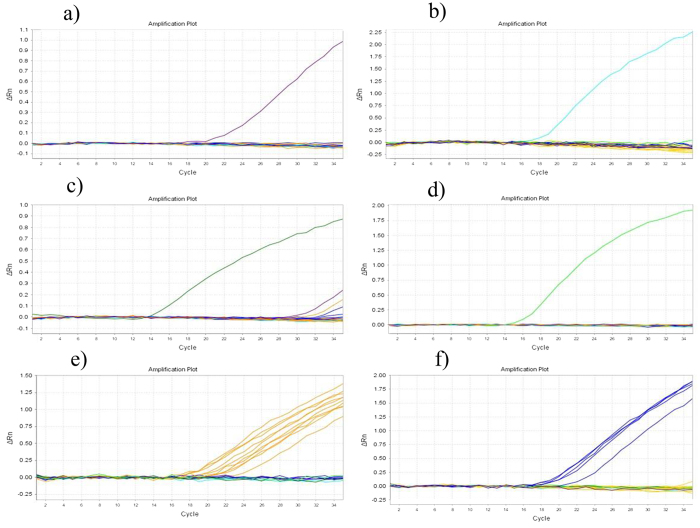
Validating species specificity of the TaqMan probe and primer sets for six *Tribolium species*. (**a**) 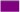 *T. destructor*. (**b**) 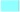 *T. brevicornis*. (**c**) 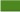 *T. madens*. (**d**) 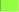 *T. freemani*. (**e**) 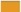 *T. castaneum*. (**f**) 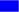 *T. confusum*.

Six TaqMan probe and primer pairs were selected for the reliable identification of six *Tribolium* species and are listed in Table 5. These primer and probe sets were designed to identify different *Tribolium* species regardless of their life stage. The results clearly demonstrated that each primer and probe set produced a species-specific band without any nonspecific bands (Fig. 6).

**Figure 7:**
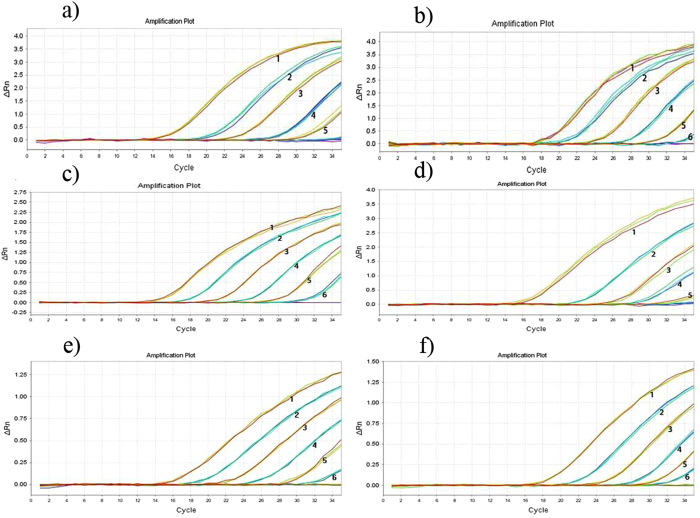
Sensitivity test for specific TaqMan probe and primer sets for six *Tribolium* species. (**a**) *T. destructor*. (**b**) *T. brevicornis*. (**c**) *T. madens*. (**d**) *T. freemani*. (**e**) *T. castaneum*. (**f**) *T. confusum*. Note: 1: 100 ng; 2: 10 ng; 3: 1 ng; 4: 0.1 ng; 5: 0.01 ng; 6: 0.001 ng (DNA template concentrations).

In the six *Tribolium* species, template concentrations lower than 0.01 ng μl^−1^ noticeably decreased the intensity of the visualized bands. Finally, the lower limit for detection was set at 0.01 ng μl^−1^ for *T. destructor* and *T. freeman* and 0.001 ng μl^−1^ for *T. castaneum, T. confusum, T. destructor* and *T. madens* (Fig. 7).

## Discussion

Molecular biology techniques have been increasingly applied to species identification. In our study, a series of experiments based on DNA barcoding, species-specific primers were performed to identify six species of the genus *Tribolium*. The results showed that DNA barcoding technology, species-specific PCR and real-time PCR are useful for the rapid and accurate identification of six *Tribolium* species. Our data demonstrated that regardless of whether the specimen is an adult, larva or pupa of *Tribolium*, it is possible to extract sufficient DNA for DNA barcoding, species-specific PCR and real-time PCR for identification of the sample. We speculate that the universal primers for the mtDNA COI gene, the species-specific primers, and the TaqMan probe and primers sets for *Tribolium* can feasibly identify all of the ontogenetic stages.

The COI gene provides a robust DNA barcode for identifying the six species of stored-product *Tribolium* with non-overlapping genetic distances between intra- and inter-specific samples (Fig. 1a). The genetic distance between sequences provides an approach for ‘DNA barcode’ evaluation^19^. A favourable DNA barcode should have a higher divergence between species than within species. For COI, the suggested standard divergence threshold value is ten times (10X) the mean intra-specific variation^20^. In this study, the ratio between species is thirty nine times (39X) the within species variation. The NJ tree organized all of the six species determined by morphology by forming robust clades.

The PCR assay with species-specific primers clearly demonstrated that each primer pair produced a species-specific band without any nonspecific bands (Fig. 4). Compared with DNA barcoding technology, species-specific PCR does not require sequencing, only routine laboratory techniques such as DNA extraction, PCR and electrophoresis, so this method is convenient for any quarantine laboratory. The same PCR assay with species-specific primers can be performed within 3 h using unknown *Tribolium* species DNA.

Six probe and primer sets were designed and selected for the real-time PCR method, which was also able to distinguish the six *Tribolium* species. Compared with traditional PCR, the real-time PCR method can be assessed directly without melting curve analysis. The amount of fluorescence generated during the reaction directly reflects the number of amplicons in real-time gene copies. However, this method also has drawbacks, e.g., the relatively high price of real-time instruments, reaction kits and TaqMan probes.

Future work should focus on collecting more samples, screening commonly used molecular markers and developing multiplex PCR. In this study, three *Tribolium* species, *T. destructor*, *T. brevicornis* and *T. madens*, are only distinct populations geographically. Meanwhile, *T. parallelus*, *T. thusa*, and especially *T. audax* Halstead in North America and *T. anaphe* Hinton in central Africa^4^ have not been collected, although attempts to acquire these samples were made. Next, internally transcribed spacer (ITS) can possibly be used for *Tribolium* species identification, despite the COI gene providing a robust DNA barcode for identifying the six species of *Tribolium* stored-product pests. Finally, species-specific primers and real-time PCR have the potential to be implemented in multiplex PCR.

## Additional Information

**How to cite this article**: Zhang, T. *et al*. DNA barcoding, species-specific PCR and real-time PCR techniques for the identification of six *Tribolium* pests of stored products. *Sci. Rep*. **6**, 28494; doi: 10.1038/srep28494 (2016).

## Figures and Tables

**Table 1:** Specimens of *Tribolium* used in the study.

Species	Collection locality	Accession number
*T. destructor*	Prague, Czech Republic	KP892664
*T. brevicornis*	York, the United Kingdom	KP892667
*T. madens*	Kansas, the United States	KP892665
*T. freemani*	Kansas, the United States	KP892666
*T. castaneum*	Henan, P. R. China	KP892686, KP892687, KP892688
	Guangxi, P. R. China	KP892680, KP892681
	Guangdong, P. R. China	KP892682, KP892683
	Xingjiang, P. R. China	KP892684, KP892685
	Prague, Czech Republic	KP892674
	Rakovník, Czech Republic	KP892675
	Osijek, Croatia	KP892677
	Bordeaux, France	KP892676
	Kansas, the United States	KP892678, KP892679
*T. confusum*	Prague, Czech Republic	KP892668
	Herink, Czech Republic	KP892669
	Kyjov, Czech Republic	KP892670
	Bordeaux, France	KP892671
	Kansas, the United States	KP892672

**Table 2:** The information of COI gene sequences downloaded from GenBank used in this study.

Species	Accesssion No.
*T. destructor*	FJ743723
*T. brevicornis*	FJ743722
*T. madens*	FJ743721
*T. freemani*	FJ743724
*T. castaneum*	KJ003352, KM207082, KM439779
*T. confusum*	FJ743725, KJ964296

**Table 3:** The intra- and inter-specific Kimura 2-parameter divergence values (%) of COI gene.

Species	Intra	Intra	Intra	Inter	Inter	Inter
	Average	Min	Max	Average	Min	Max
*T. destructor*	0.00	0.00	0.00	20.18	18.66	21.38
*T. brevicornis*	0.92	0.00	0.92	20.43	18.00	22.28
*T. madens*	0.00	0.00	0.00	19.23	18.62	20.30
*T. freemani*	0.00	0.00	0.00	17.89	16.34	22.28
*T. castaneum*	0.57	0.00	1.86	19.74	16.34	21.39
*T. confusum*	0.04	0.00	0.15	19.69	18.00	20.46
All	0.50	0.00	1.86	19.61	16.34	22.28

**Table 4:** List of the specific primers of the 6 storage *Tribolium* species.

Species	Primers’ name	Sequence(5′-3′)	bp	Tm(°C)	Product length (bp)
*T.destructor*	Tde25F20	ATGAGCAGGAATAGTTGGTA	20	51.6	450
	Tde451R24	ATAGGTCGTATATTGATTACTGTG	24	51.7	
*T. brevicornis*	Tbr63F23	TTCGAGCAGAATTAGGTAATCCC	23	55.8	354
	Tbr394R23	TTCCTGCTAAATGTAATCTAAAG	23	50.7	
*T. madens*	Tma41F22	GGAACCTCTTTAAGATTATTAG	22	49.7	490
	Tma508R23	TTGCTGTAATTACCACAGCTCAG	23	57.2	
*T. freemani*	Tfr379F22	CGTAGATTTAGCAATTTTCAGG	22	53.3	170
	Tfr526R23	GTGAAAGAAGTAGAAGAATAGCG	23	52.2	
*T. castaneum*	Tca33F26	GAATAGTAGGCACTTCATTAAGACTC	26	56.3	337
	Tca346R24	CCATGTGCAATGTTTGATGAGAGG	24	57.9	
*T. confusum*	Tco261F23	GGCTCCTGCCACCCTCATTAAGA	23	61.7	238
	Tco474R25	GGTATTCGTTCAAATGATATTCCTG	25	55.7	

**Table 5:** List of TaqMan probes and primers of the 6 storage *Tribolium* species.

Secies	Primers andProbes	Sequence 5′-3′	bp	Tm(°C)	Product (bp)
*T.destructor*	TdeF	CGTACAGAACTAGGAAAC	18	58.1	116
	TdeR	CCGATTATTATAGGTATTACTATG	24	57.8	
	TdeP	FAM-TCCTTAATCGGGAATGACCAAAT-BHQ	23	65	
*T. brevicornis*	TbrF	GAGCAGTAGCAATTACAG	18	58.9	84
	TbrR	TTCGGTCGGTTAATAATATAG	21	58.7	
	TbrP	FAM-TCACTTCCAGTGTTAGCCGGTG-BHQ	22	69.6	
*T. madens*	TmaF	TCCTGGTTCTCTAATTGG	18	59.3	138
	TmaR	GCTCCTAGTATAAGTGGAA	19	59.1	
	TmaP	FAM-AATGTAATTGTCACAGCCCATGC-BHQ	23	67.1	
*T. freemani*	TfrF	CGTAGATTTAGCAATTTTCAGG	22	61.7	169
	TfrR	TGAAAGAAGTAGAAGAATAGCG	22	61.9	
	TfrP	FAM-AGCTGGTATCTCATCAATTCTTGGAGC-BHQ	27	69.8	
*T. castaneum*	TcaF	GATCCTCTGTTGATCTTG	18	58.1	183
	TcaR	CAGGAAGAGATAAGAGAAG	19	57.5	
	TcaP	FAM-TCTGGGAGCAGTTAATTTCATTACAAC-BHQ	27	66.8	
*T. confusum*	TcoF	CAGGATGAACTGTTTACC	18	58.7	151
	TcoR	GTAGGTCGTATATTAATTACTG	22	57.3	
	ToP	FAM-ATCATCTAATATCGCTCACGGAGGAG-BHQ	26	68.6	
